# Palladium/palladium oxide coated electrospun fibers for wearable sweat pH-sensors

**DOI:** 10.1038/s41598-019-45399-2

**Published:** 2019-06-20

**Authors:** Victor C. Diculescu, Mihaela Beregoi, Alexandru Evanghelidis, Raluca F. Negrea, Nicoleta G. Apostol, Ionut Enculescu

**Affiliations:** 1 National Institute of Materials Physics Bucharest, Magurele, 077125 Romania; 2 University of Bucharest, Faculty of Physics, 405 Atomistilor Street, 077125 Magurele, Romania

**Keywords:** Sensors, Sensors and biosensors

## Abstract

The work describes the development of a flexible, hydrogel embedded pH-sensor that can be integrated in inexpensive wearable and non-invasive devices at epidermal level for electrochemical quantification of H^+^ ions in sweat. Such a device can be useful for swift, real time diagnosis and for monitoring specific conditions. The sensors’ working electrodes are flexible poly(methyl methacrylate) electrospun fibers coated with a thin gold layer and electrochemically functionalized with nanostructured palladium/palladium oxide. The response to H^+^ ions is investigated by cyclic voltammetry and electrochemical impedance spectroscopy while open circuit potential measurements show a sensitivity of aprox. −59 mV per pH unit. The modification of the sensing interface upon basic and acid treatment is characterized by scanning and transmission electron microscopy and the chemical composition by X-ray photoelectron spectroscopy. In order to demonstrate the functionality of the pH-sensor at epidermal level, as a wearable device, the palladium/palladium oxide working electrode and silver/silver chloride reference electrode are embedded within a pad of polyacrylamide hydrogel and measurements in artificial sweat over a broad pH range were performed. Sensitivity up to −28 mV/pH unit, response time below 30 s, temperature dependence of approx. 1 mV/°C as well as the minimum volume to which the sensor responses of 250 nanoliters were obtained for this device. The proposed configuration represents a viable alternative making use of low-cost and fast fabrication processes and materials.

## Introduction

The steep development in information and communication technologies, which includes small and powerful portable devices and ubiquitous broadband communication, opened up new pathways in personalized healthcare^1^. The possibility of continuous, real time health monitoring represents nowadays an achievable goal and consequently numerous efforts are dedicated to the development of new wearable sensors and medical devices^2–4^.

Sweat is composed by a number of biomarkers including ions (H^+^, Na^+^, K^+^, NH_4_^+^, Cl^−^), small molecules (cortisol, urea, lactate, glucose, uric acid etc.), and even peptides or small proteins (neuropeptides and cytokines) providing a very complex matrix^5^ that contains information of physiological processes and vital signs. Thus, monitoring sweat is a valuable health monitoring and diagnostic tool. In particular, sweat pH gives important information about sweat rate, dehydration but also on the physiological status of the epidermis which may be directly related to eventual wound healing processes. Wearable sensors and biosensors with different architectures^6,7^ for monitoring transcutaneous oxygen and humidity, Na^+^, pH, NH^4+^, K^+^ and Cl^−^ or lactate and glucose have been described^1,4,8–12^. Skin patches are natural first choices in terms of constructive principle^13^. Although low cost, wearable health monitoring devices are already available on the market^14^, a fully wearable skin placed sensor which can read information ranging from temperature to sweat composition or electrical signals is not easy to develop^15^ Sensors based on electrical measurements are among the most difficult to build since the electrodes they incorporate should be biocompatible, of low mass and highly flexible or even foldable^15,16^.

The field of flexible electrodes in the broad subject of flexible electronics^17^ is continuously investigated for numerous purposes^18^. Flexible printed circuit boards are largely used^1,18^ but polymer foils and/or meshes of microscopic metallic fibers and nanowire networks represents valuable alternatives^19^. Such electrode systems could be excellent candidates for skin patch sensors for quantification of biomarkers in sweat, since they provide all the necessary functionality and properties including here excellent electrical characteristics and flexibility.

Metallic highly conductive microscopic meshes can be obtained in several ways, in most cases through low cost and scalable methods^20^. As an example, electrospinning is a technique that allows obtaining submicronic polymeric fibers^21^ which, further, can be easily coated with metal layers in order to obtain a conductive path and be attached to almost any type of substrate for fabrication of flexible electrodes.

In this context, this work describes the fabrication of flexible and conformable pH sensors based on metallized electrospun polymeric fibers. A palladium/palladium oxide system was chosen as sensing interface due to its pH sensitivity^22,23^ but also due to its electrocatalytic properties^24–27^. In order to demonstrate the functionality of the sensor for measurements at skin’s surface, the contacting of the sensing interface with the biomaterial to be measured was achieved in a non-invasive way by using a hydrogel film thin enough in order to provide a short path to the electronic part components. The performance of the sensor was investigated in different media including artificial sweat. Sensitivity, response time, temperature dependence volume of detection as well as mechanical stability were investigated. The proposed configuration is original and represents an alternative to other/more complicated architectures since it makes use of a fast fabrication process and low-cost materials providing the possibility of disposable sensors. Thus, electrochemical wearable sensors for sweat monitoring are usually based on microelectrodes fabricated by employing lithographical techniques^1^. The present approach maximizes important parameters such as specific surface and flexibility.

## Materials and Methos

### Materials and reagents

Poly(methyl methacrylate) (PMMA), acryalamide (AA), N,N-methylenebisacrylamide (MBA), 2-hydroxy-4′-(2-hydroxyethoxy)-2-methylpropiophenone (Irgacure 2959), *N*,*N*-dimethylformamide (DMF), HCl and PdCl_2_ were obtained from Sigma-Aldrich and used without purification. Polyethylene terephthalate (PET) (having the thickness of 0.038 mm) foils were purchased from Good Fellow.

Buffer supporting electrolyte solutions of acetate (HAcO + NaAcO pH 4.5 and 5.6), phosphate (NaH_2_PO_4_ + Na_2_HPO_4_ pH 6.8 and 7.8) and borate (Na_2_B_4_O_7_ + NaOH pH 9.2), were prepared using analytical grade reagents and deionized water from a Millipore Milli-Q system (conductivity ≤0.1 µS cm^−1^). The pH measurements were carried out with a Hannah pH-meter. All experiments were done at room temperature (25 ± 1 °C).

Artificial sweat was prepared taking into consideration the European Standard EN1811:2012 and other previous reports^28^. Briefly, 0.5% of NaCl, 0.1% of KCl, 0.1% lactic acid, and 0.1% urea and 0.01% glucose (w/v) in deionized water.

### pH sensor

**Figure 1:**
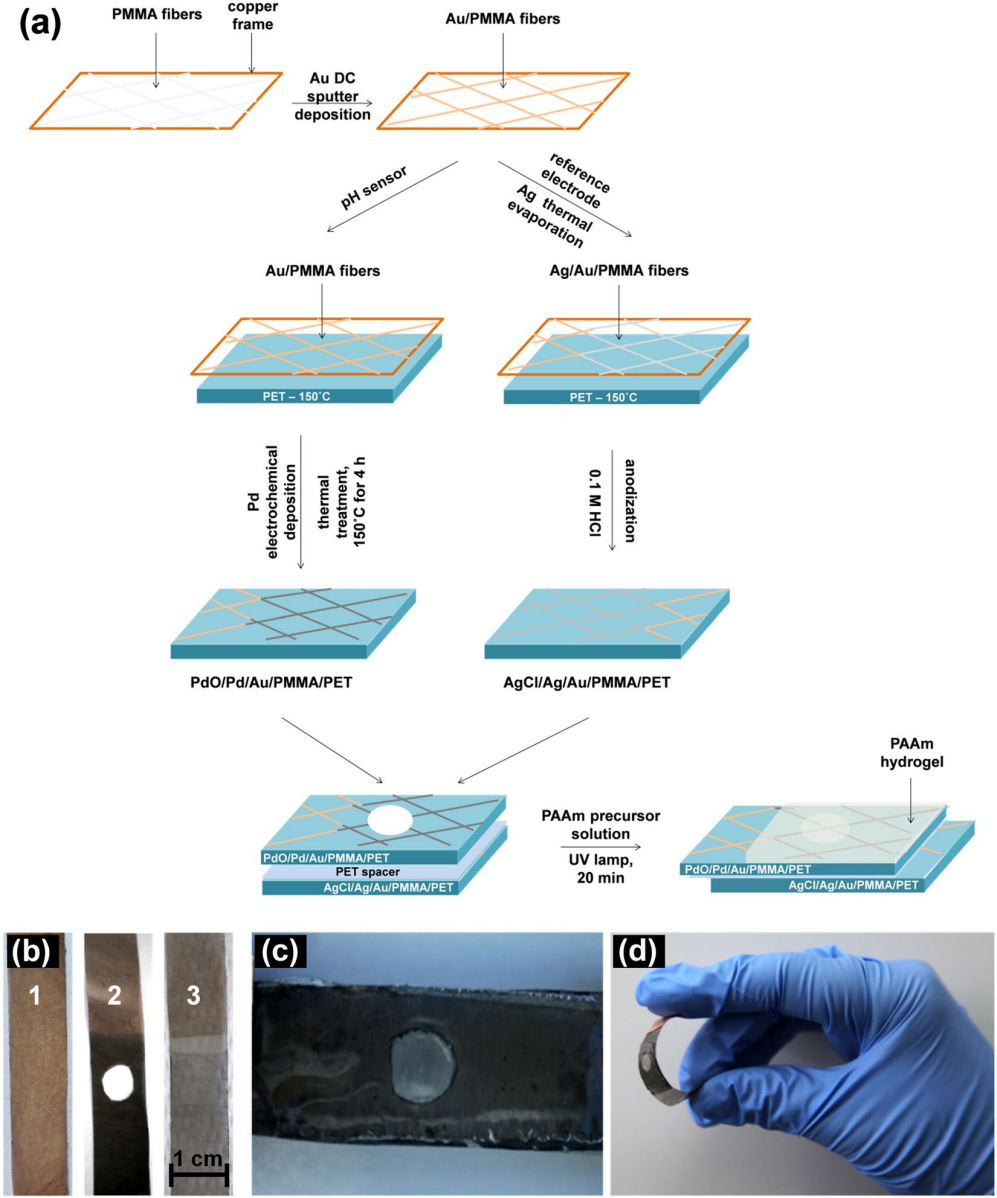
(**a**) Schematic representation of the steps involved in the fabrication process of the electrodes. Photographs of: (**b**) 1 Au/PMMA/PET, 2 Ag-Cl/Ag/Au/PMMA/PET reference electrode and 3 PdO/Pd/Au/PMMA/PET pH sensor, (**c**) hydrogel embedded pH-sensor and (**d**) its flexibility.

The fabrication of the sensor involved several steps, which are schematically presented in Fig. 1(a). First, polymer fibers were obtained by electrospinning a 10% PMMA solution in optimized experimental conditions^20^. Submicronic fibers of PMMA as nonwoven meshes were obtained with the solution flux of 0.5 mL/h under 15 kV applied potential on the spinneret. The fibers were collected during 10 min on cooper frames placed at 15 cm away from the spinneret. In a second step, PMMA fiber mats were covered by magnetron sputtering with 200 nm gold layer (Au/PMMA) in order to make them conductive. The Au/PMMA fibers were thermally attached onto flexible PET substrates by heating the ensemble at 150 °C, Fig. 1(b) no. 1.

The deposition of the sensing Pd layer was performed into a three electrode cell, using the Au/PMMA/PET as working, platinum plate as counter and saturated calomel electrode (SCE) as reference electrode. The deposition of Pd was performed during 2 h in 3 mM PdCl_2_ in 0.1 M HCl at an applied potential of +0.10 V^26^, leading to Pd/Au/PMMA/PET electrode. Then, the Pd/Au/PMMA/PET was annealed in air for 4 h at 150 °C in order to oxidize the Pd layer (this was found to be an optimal temperature since lower temperatures do not lead to the formation of an oxide layer while higher teperatures damage the device by damaging the structure of the fiber). This procedure led to the PdO/Pd/Au/PMMA/PET pH sensor, Fig. 1(b) no. 2.

A similar procedure was followed in order to obtain PdO/Pd/Au/Ti/SiO_2_/Si electrodes for Transmission electron microscopy (TEM) and X-rays photoelectron spectroscopy (XPS) analysis.

### Proof of concept

To demonstrate the functionality of the pH-sensor at epidermal level, AgCl/Ag/Au/PMMA/PET reference electrodes were also produced, Fig. 1(b) no. 3. Gold-coated nets were half-coated with a thin silver layer (Ag/Au/PMMA) by thermal evaporation. Then, fibers were attached on PET substrate (Ag/Au/PMMA/PET) and the assembly was anodized in 0.1 M HCl at +1.00 V during 2 min.

The PdO/Pd/Au/PMMA/PET pH sensor was placed on top of the AgCl/Ag/Au/PMMA/PET reference electrode in an anti-parallel configuration so that the extremity of each electrode provided the electric contacts to the potentiostat, Fig. 1(a). An aperture in the PdO/Pd layer was created, Fig. 1(b), to allow the electrochemical communication between the pH sensor and the reference electrodes. Also, a perforated PET spacer was used in between both electrodes for best isolation and overcome any short-circuit of the system. In the last step, the pH sensor surface was coated with the hydrogel precursor aqueous solution which consisted of 5% (w/w) AA and cross-linking agent MBA at a molar ratio of 100:1. The polymerization of AA was initiated by 0.8% w/w Irgacure 2959 under UV lamp irradiation with the wavelength required for initiator decomposition. The resulting hydrogels were washed with deionized water and dried overnight in air, at room temperature. This process led to the final flexible pH sensor, Fig. 1(c,d).

The sensor response was investigated by measuring the open circuit potential (OCP) variation with time and with the pH of the environment. For this, 100 µL of artificial sweat was dropped on the PAAm-covered sensor surface. The sensor was incubated and the hydrogel allowed hydrating during 20 min. Then, the excess artificial sweat solution was removed with absorbent paper and OCP measured during 2 min or until stabilization. The procedure was repeated using the same sensor for the measurement of different pH values of the artificial sweat.

### Electrodes characterization

The characterization of the pH sensor and its constituents was performed after each fabrication step. The morphology of the electrospun fibers before and after sputtering the Au and after electrochemical deposition of the Pd layer or thermal treatment for the formation of PdO was investigated by scanning (SEM) and transmission electron microscopy (TEM). Also, X-rays photoelectron spectroscopy (XPS) analysis was employed to determine the formation of PdO and its behavior under the modification of pH. Electrochemical characterization of the electrodes was also performed.

#### Scanning electron microscopy (SEM)

A Zeiss Evo 50 XVP Scanning Electron Microscope was used for the morphological characterization of the Au/PMMA/PET and working PdO/Pd/Au/PMMA/PET electrodes. The SEM images were acquired at the magnifications of 5 and 50 kV, at a working distance of 15 mm, with an accelerating voltage of 20 kV and a spot size of 300.

#### Transmission electron microscopy (TEM)

The TEM investigations have been performed on a Cs probe-corrected JEM ARM 200 F analytical electron microscope operated at 200 kV. STEM images have been recorded using the High-Angle Annular Dark Field (HAADF) detector. Images processing have been made using specialized routines under Gatan Digital Micrograph. Cross-section TEM specimens have been prepared from the samples by mechanical polishing down to ca. 30 µm, followed by ion milling in a Gatan PIPS machine at 4 kV accelerating voltage and 7° incidence angle. Low-voltage (2 kV) milling was used as final ion polishing stage in order to reduce the amorphous surface layer enveloping the specimen.

#### X-ray photoelectron spectroscopy (XPS)

X-Ray Photoelectron Spectroscopy (XPS) was performed in an AXIS Ultra DLD (Kratos Surface Analysis) setup equipped with an 180° hemispherical analyzer, using Al K_α1_ (1486.74 eV) radiation produced by a monochromatized X-Ray source at operating power of 300 W (15 kV × 20 mA). The base pressure in the analysis chamber was at least 1.0 × 10^−8^ mbar. Partial charge compensation was reached by using a flood gun operating at 1.52 A filament current, 2.73 V charge balance, 2.02 V filament bias. The survey spectra were recorded using Hybrid lens mode, 80 eV pass energy, slot aperture, and high resolution core level spectra were recorded using Hybrid lens mode, 20 eV pass energy slot aperture. The core level spectra have been deconvoluted using Voigt profiles, based on previously described method^29–37^.

#### Electrochemistry

The electrochemical (EC) characterization of the sensor and its components was performed using a VoltaLab PGZ100 potentiostat. The experimental parameters for the cyclic voltammetry (CV) were: scan rate *v* = 100 mV s^−1^ at a step potential of 2 mV. The open circuit potential (*OCP*) values of the PdO/Pd/Au/PMMA/PET pH sensor were measured *vs*. the saturated calomel reference electrode (SCE) until stable values were recorded. The electrochemical impedance spectroscopy (EIS) measurements were carried out using a perturbation of 10 mV. The data was collected for 30 harmonic frequencies from 10 kHz to 0.01 Hz, at 5 steps/decade, and different polarization potentials corresponding to open circuit potential (*OCP*) values. The impedance spectra were analyzed by fitting using ZView software (Scribner Associates, USA) to the Randles-type equivalent electrical circuit.

## Results and Discussions

The fabrication of the sensor involves the steps schematically presented in Fig. 1. First, polymer fibers were obtained by electrospinning a poly(methyl methacrylate) (PMMA) solution. In a second step, the PMMA fiber mats were covered by magnetron sputtering with a gold layer (Au/PMMA). Then, the Au/PMMA fibers were thermally attached onto flexible PET substrates in order to obtain the Au/PMMA/PET electrodes. pH sensors were obtained through the electrochemical deposition of Pd metal on the Au/PMMA/PET. Then, the Pd/Au/PMMA/PET was annealed in air in order to oxidize the surface of the Pd layer and produce the working electrode PdO/Pd/Au/PMMA/PET. In a similar way, PdO/Pd/Au/Ti/SiO_2_/Si electrodes were obtained for material characterization.

### Electrodes characterization

#### Morphological

**Figure 2:**
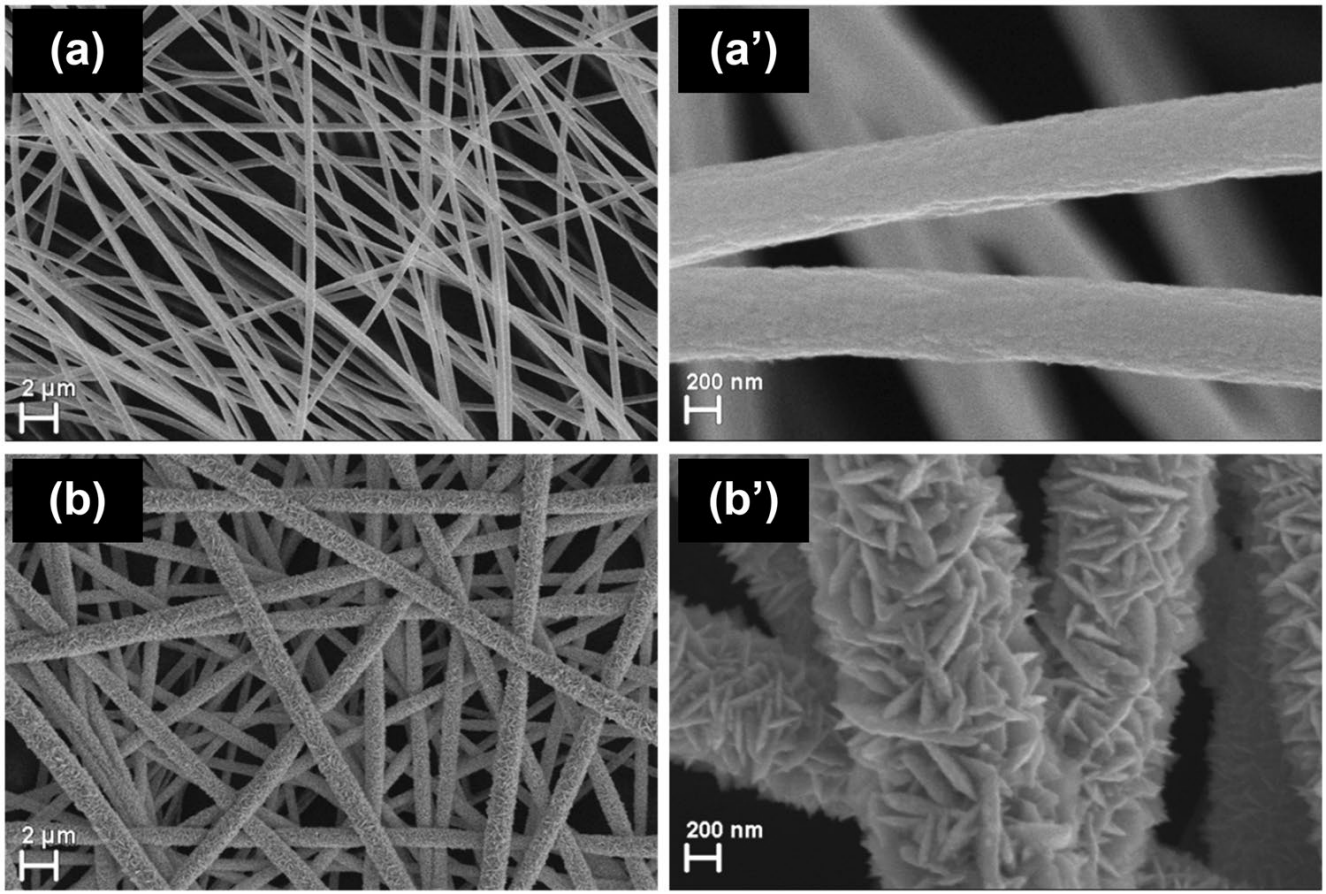
SEM images of (**a**,**a’**) Au/PMMA/PET and (**b**,**b’**) PdO/Pd/Au/PMMA/PET electrodes.

Figure 2 presents the SEM images of Au/PMMA/PET and PdO/Pd/Au/PMMA/PET electrodes. The PMMA fibers were covered with a smooth and homogeneous gold layer and showed an average diameter size of about 0.5 μm, Fig. 2(a,a’). Further, after Pd electrochemical deposition and thermal treatment, fibers of about 1 µm with a flake-like structure and acicular extremities were observed, Fig. 2(b,b’). A hierarchical architecture of individual nanostructures leads to a continuous mixt layer of Pd and PdO.

The above mentioned dimensions were chosen for two reasons: (i) straightforward approach to produce fiber webs with the desired properties (much thinner fibers would require better control of fabrication conditions and would result in lower throughput) and (ii) high mechanical resistance (thicker metal/metal oxide layer would result in brittle fibers). In present experimental conditions fiber thickness is determined by polymer solution concentration, temperature and humidity of the process its uniformity varying with a low value (100 nm differences at most). Moreover, electrodes fabricated with fibers of slightly different dimensions did not show any significant differences. However, for simplicity, the electrospinning process was carried out at room temperature and 40–60% RH.

**Figure 3:**
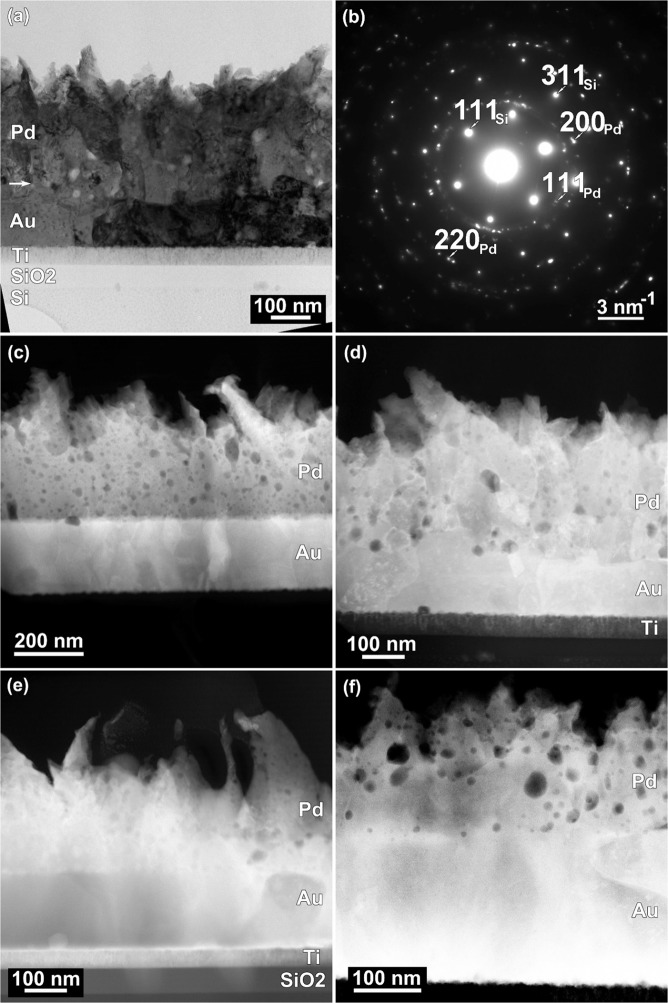
(**a**) TEM image at low-magnification of PdO/Pd/Au/Ti/SiO2/Si electrodes. (**b**) SAED pattern corresponding to TEM image (**a**). (**c**,**f**) HAADF-STEM images at low-magnification showing the morphology of Pd layers in PdO/Pd/Au/Ti/SiO_2_/Si electrodes after: (**c**) EC deposition, (**d**) thermal, (**e**) basic and (**f**) acid treatment.

TEM images, Fig. 3(a), showed the morphology of the Pd layer. In this case PdO/Pd/Au/Ti/SiO2/Si sensors were fabricated on silicon wafers due to necessity of the milling/polishing stage in order to reduce the amorphous surface layer (*Section 4*. *Experimental*. *Transmission electron microscopy*).

The PdO layer appeared with a disordered columnar growth and a flake-like structure with acicular extremities, as in the case of SEM images. For all samples, the SAED patterns, Fig. 3(b), revealed the cubic phase of Pd with space group Fm3m and a = b = c = 3.86 Å, with no crystalline texture. Diffraction spots corresponding to Pd film and to Si and Au from substrate were identified in the SAED patterns.

HAADF-STEM images in Fig. 3(c–f) correspond to the as-deposited Pd layer, Pd layer after thermal treatment at 150 °C for production of the PdO and basic or acid treatment during 12 h in 0.1 M NaOH or HCl. The mass-thickness contrast from HAADF-STEM images showed a porous structure of the Pd layer, with different density and diameters of pores, which almost completely disappeared after an alkaline treatment. Nevertheless, the thickness of the Pd/PdO layer was strongly affected by an acid treatment decreasing from about 400 nm after electrochemical deposition and thermal treatment to approx. 200 nm. The flake-like, acicular shape of the nanostructured Pd/PdO layer was still observed.

#### X-ray photoelectron spectroscopy; Chemical composition

**Figure 4:**
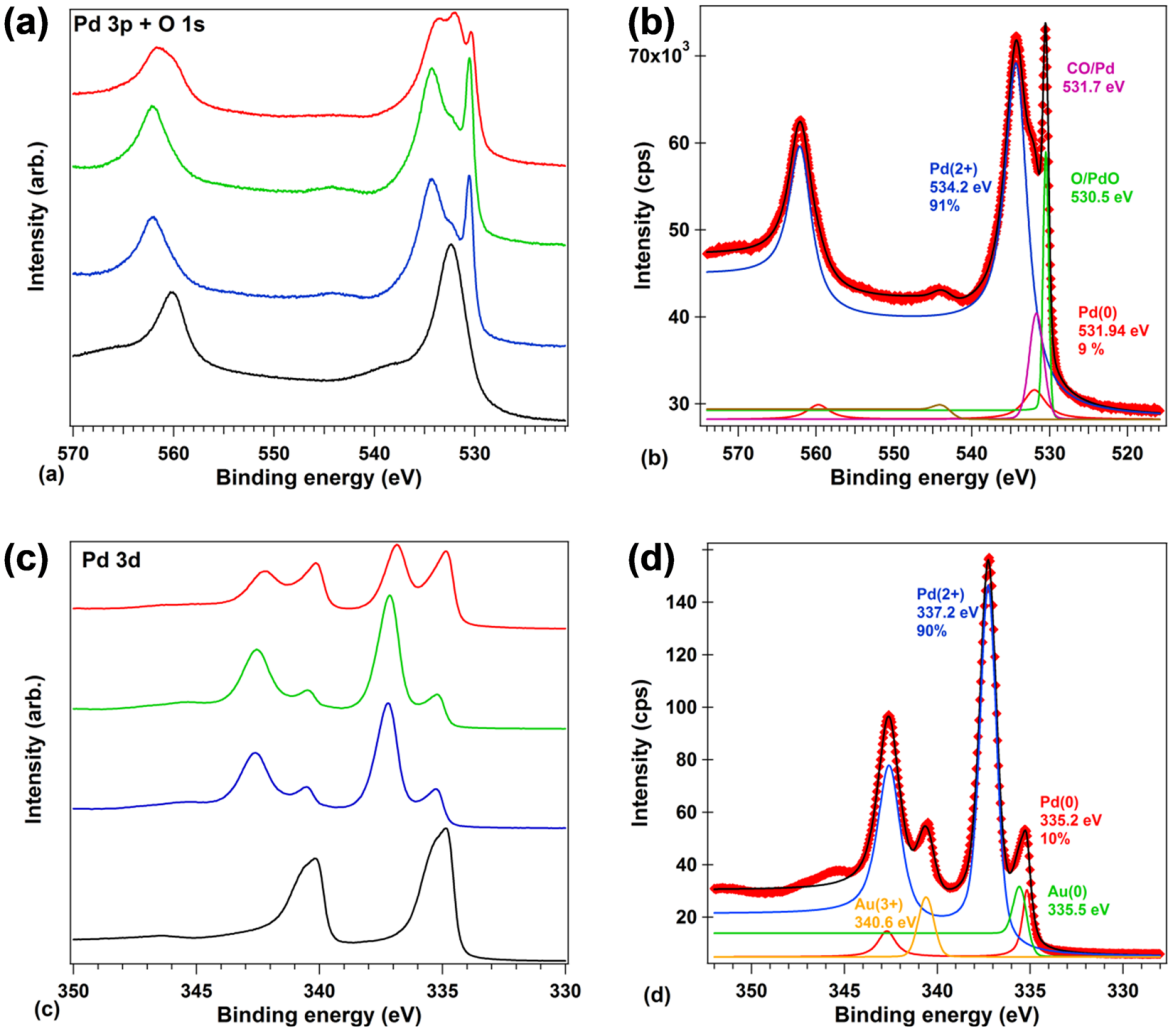
XPS spectra of (**a**,**b**) Pd 3p and O 1 s, (**c**,**d**) Pd 3d recorded on PdO/Pd/Au/Ti/SiO2/Si electrodes after (black) EC deposition, (blue) thermal, (green) basic and (red) acid treatment. (**b**,**d**) Deconvolution of the signals on the spectra recorded after thermal treatment.

The chemical composition at the surface of PdO/Pd/Au/Ti/SiO_2_/Si electrodes was characterized by X-ray photoelectron spectroscopy (XPS), after electrochemical deposition of the Pd layer, thermal treatment at 150 °C for obtaining the PdO and basic or acid treatment during 12 h in 0.1 M NaOH or HCl. The XPS spectra recorded on energy ranges corresponding to Pd 3p – O 1 s and Pd 3d are represented in, Fig. 4. Between 520 and 570 eV, Fig. 4(a), after EC deposition of the Pd layer, the Pd/Au/PMMA/PET showed two main peaks at 560.0 and 532.6 eV corresponding to 3p_1/2_ and 3p_3/2_ of Pd(0), respectively.

The detailed data analysis by ‘deconvolution’ revealed also the O 1 s peak at binding energy 531.6 eV, mixed in the Pd 3p3/2 line. This O 1 s component is attributed to CO adsorbed on Pd. After thermal treatment and formation of the PdO layer two additional peaks occurred in the Pd 3p3/2 region at 534.2 and 530.5 eV. The deconvolution of the spectra allowed identifying these signals with an increased amount of Pd(2+) and of oxygen, respectively, proving the existence of the PdO layer after thermal treatment, Fig. 4(b). After basic treatment, no significant modification in the PdO layer was registered, Figure 4(a) and Table 1. But a different situation was found after acid treatment where the percentage of Pd(2+) and oxygen decreased together with the increase of the Pd(0), Table 1 and Fig. 4(a), in agreement with the HAADF-STEM experiments and with the electrochemical reaction in Eq. (1):

1 $${\rm{PdO}}+2{{\rm{e}}}^{-}+{{\rm{2H}}}^{+}\to {\rm{Pd}}+{{\rm{H}}}_{2}{\rm{O}}\,(\,+\,0.790\,{\rm{V}})$$

**Table 1:** Contents of Pd(0) and Pd(2+) measured by XPS, Fig. 4, at the surface of PdO/Pd/Au/Ti/SiO_2_/Si electrodes after electrochemical deposition of Pd layer, thermal treatment at 150 °C during 4 h and acid (0.1 M HCl) or basic (0.1 M NaOH) treatment during 12 h.

	Pd 3p		Pd 3d	
	Pd(0) [%]	Pd(2+) [%]	Pd(0) [%]	Pd(2+) [%]
EC deposition	92	8	63	37
thermal treatment	9	91	10	90
basic treatment	8	92	9	91
acid treatment	30	70	34	66

A similar situation was encountered on the energy range between 330 and 350 eV, Fig. 4(c), correspondent to Pd (0) 3d orbitals. The Au 4d_5/2_ levels from the substrate also occurred in this region. Briefly, after thermal treatment the formation of the PdO layer was proved by the increase amount of Pd(2+) at 337.2 eV. The basic treatment did not affect the PdO layer, contrary to acid treatment where the percentage of Pd(2+) decreased together with the increase of the Pd(0), Table 1 and Fig. 4(d).

#### Electrochemical

**Figure 5:**
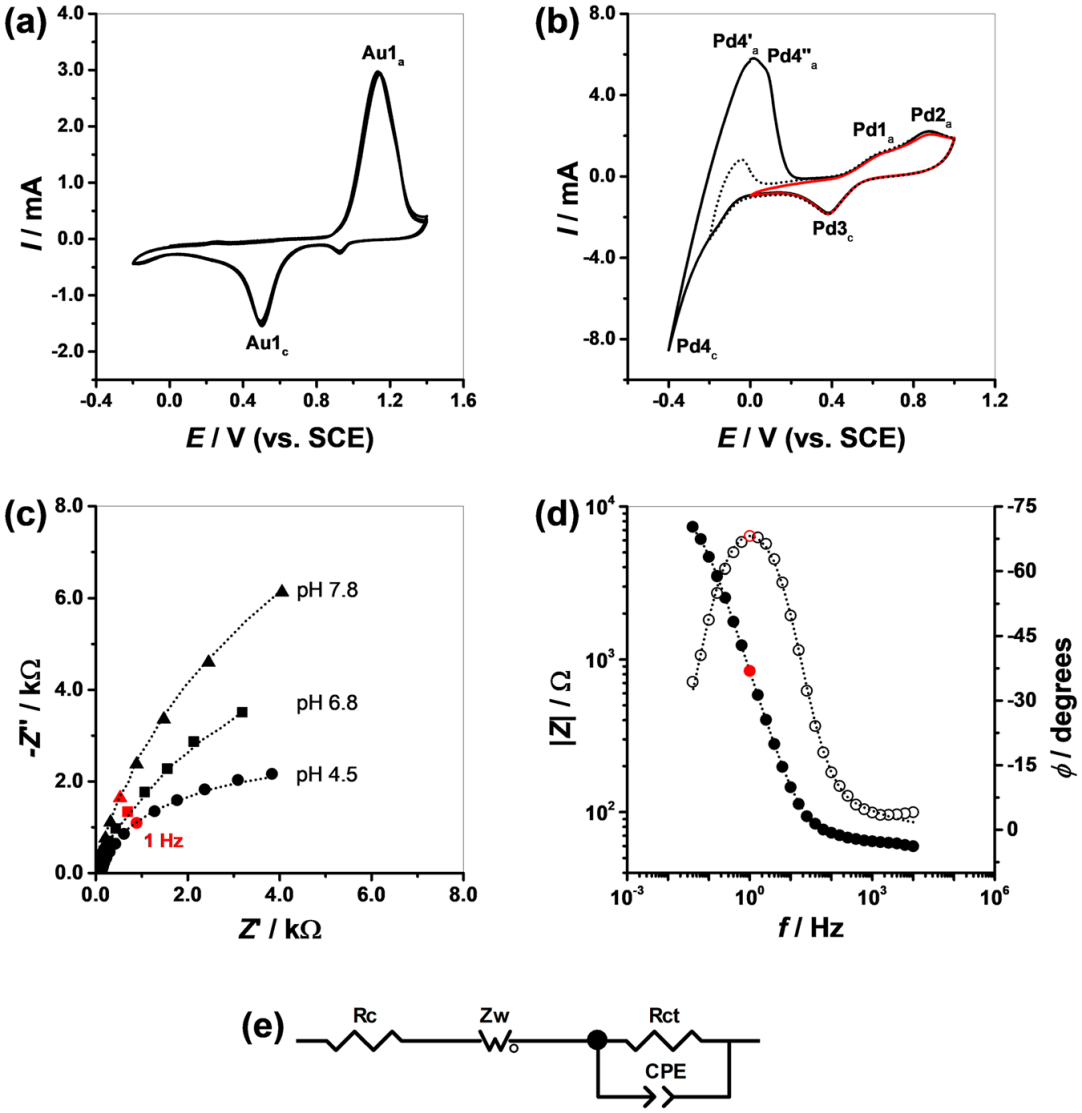
(**a**,**b**) Cyclic voltammograms in 0.1 M H_2_SO_4_ at *v* = 100 mV s^−1^ of: (**a**) Au/PMMA/PET and (**b**) PdO/Pd/Au/PMMA/PET. (**c**,**d**) EIS spectra of Pd-PdO/Au/PMMA/PET: (**c**) Nyquist diagram representing the imaginary component –*Z*” vs. the real component *Z*’ of the total impedance *Z* recorded in electrolytes with different pH values. (**d**) Bode diagram representing |*Z*| and phase angle *φ* vs. frequency *f* recorded in pH 4.5. (**e**) Schematic representation of the circuit used to fit the experimental EIS data points. The dotted curves represent the fitting results of the experimental data points with the equivalent circuit.

The electrodes were characterized after each fabrication step by electrochemical methods. Cyclic voltammetry and electrochemical impedance spectroscopy (EIS) measurements were performed in different media with different pH values, Fig. 5.

Cyclic voltammograms were initially recorded with the Au/PMMA/PET electrode in 0.1 M H_2_SO_4_. A typical behavior of the gold layer was observed, Fig. 5(a). On the positive scan of the first voltammogram the oxide formation occurred at peak Au1_a_. After changing the scan direction the cathodic peak Au1_c_ corresponded to the reduction of the oxides to metallic Au.

The deposition of the Pd layer changed this behavior, Fig. 5(b). The cyclic voltammograms recorded in the same conditions with the Pd/Au/PMMA/PET electrode showed peaks Pd1_a_ and 2_a_ corresponding to formation of oxides of palladium with oxidation states 2+ and 4+, respectively. Peak 3_c_ was due to the reduction of the oxide layer formed during the positive-going scan. On the other hand, peak 4_c_ involved the electrocatalytic reduction of H^+^ ions to hydrogen. After changing the scan direction, peaks 4_a_′ and 4_a_″ corresponded to the oxidation of the H_2_ adsorbed onto and absorbed into the Pd lattice. This voltammetric behavior is typical for palladium and has been described in detail in previous publications^24,25^.

The behavior of the Pd/Au/PMMA/PET electrode was also investigated using EIS. The spectra were recorded in electrolytes with different pH values at the corresponding open circuit potential (*vs*. SCE), Fig. 5(c,d).

The electrochemical impedance spectra always included the semi-circular part corresponding to an electron transfer process in agreement with Eq. (1). The EIS showed that, increasing the pH, both real and imaginary impedance increased.

The EIS were fitted using a Randles-type equivalent electrical circuit, Fig. 5(e), formed by the electrochemical cell resistance *R*_c_ and a parallel combination of a constant phase element *CPE*, the charge transfer resistance *R*_ct_ and a Warburg element *Z*_W_, simulating the diffusion process. The constant phase elements, defined as Eq. (2):

2 $$CPE=-\,{(Ci\omega )}^{-n}$$

is modelled as a non-ideal capacitor where the capacitance *C* describes the charge separation at the double layer interface and the *n* exponent is due to the heterogeneity of the surface. The definition of the Warburg element used is given by Eq. (3):

3 $${Z}_{{\rm{W}}}({W}_{{\rm{O}}})={R}_{{\rm{diff}}}{(i\tau \omega )}^{-{\rm{\alpha }}}{\rm{ctnh}}({[i\tau \omega ]}^{{\rm{\alpha }}})$$

where *R*_diff_ is a diffusion resistance of electroactive species, *τ* is a time constant depending on the diffusion rate (*τ* = *l*^2^/*D*, where *l* is the effective diffusion thickness, and *D* is the effective diffusion coefficient of the specie), and *α* = 0.50 for a perfect uniform flat interface. Values of *α* less than 0.50 correspond to a not uniform interface (as happens with *CPE* non-ideal capacitance when *n* < 1).

Data from analysis of EIS, Table 2, showed values of cell resistance ranging from 15 to 100 Ω and also the increase of *R*_ct_ with the pH increase as predicted by the reaction in Eq. (1).

**Table 2:** EIS data fitting results with equivalent circuit in Fig. 5(e).

pH	*OCP* [mV]	*R*_c_ [Ω]	*R*_ct_ [kΩ]	*CPE*		*Z*_W_		
				*C* [mF]	n	*W-R* [Ω]	*W-T* [s]	*W-P*
4.5	403	111	2.4	0.30	0.86	28.1	0.0218	0.40
5.6	343	57.8	4.7	0.32	0.92	30.6	0.0095	0.39
6.8	256	15.4	4.9	0.40	0.86	6.0	0.0023	0.43
7.8	199	15.4	5.7	0.57	0.88	2.8	0.0016	0.45
9.3	128	120	17.6	0.30	0.83	20.1	0.0110	0.38

*R*_c_ represents the cell resistance, *Z*_W_ the diffusion impedance, *R*_ct_ the charge transfer resistance and *CPE* the interfacial capacitance.

On the other hand, the interfacial capacitance *C* increased with pH due to the decrease of the PdO/Pd thickness as demonstrated by TEM images, whereas the heterogeneity parameter *n* remained approximately constant with values between 0.83–0.92, in agreement with the SEM and TEM results which show the roughness of the PdO/Pd film, Figs 2 and 3.

### pH response

**Figure 6:**
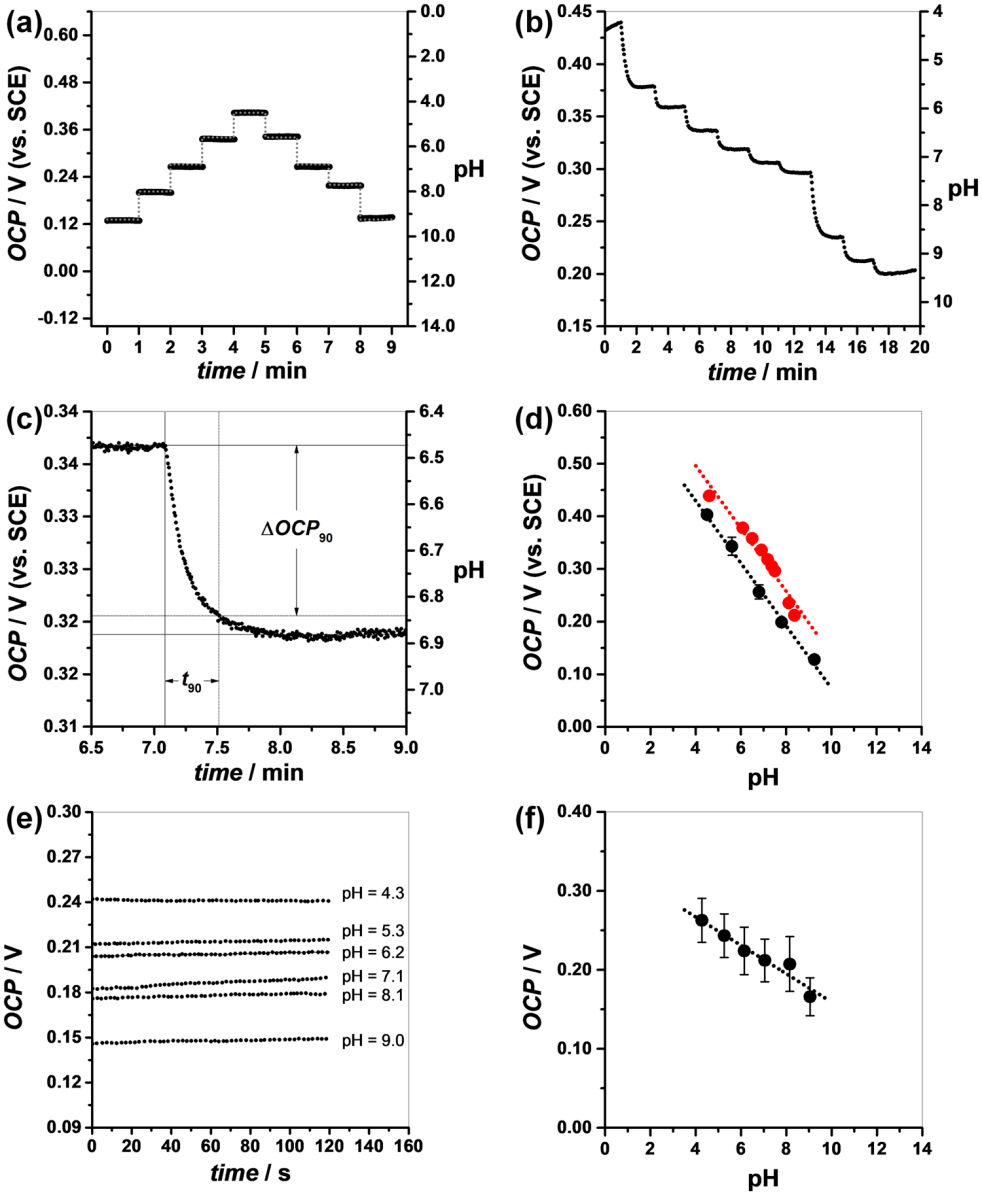
(**a**) OCP of PdO/Pd/Au/PMMA/PET function of time recorded in electrolytes with different pH values. The timescale does not take into consideration the time needed to switch between different electrolytes. (**b**) Same as (**a**) in artificial sweat. (**c**) Analysis of transition between pH 6.44 and 6.84 in artificial sweat. (**d**) Variation of OCP with the pH of the electrolyte (●) or of artificial sweat (●). (**e**) OCP of the hydrogel embedded pH sensor in artificial sweat with different pH values function of time. (**f**) Variation of OCP (n = 5) of the hydrogel embedded pH-sensor with the pH of the artificial sweat.

The pH sensitivity and measurement reversibility of the PdO/Pd/Au/PMMA/PET electrodes was confirmed by measuring the open circuit potential (*OCP*) in buffer solutions, Fig. 6(a), and/or artificial sweat, Fig. 6(b,c), during different time intervals. The electrode was tested in each buffer solution for about 60 s and then transferred into the next buffer. The *OCP* was recorded as a function of time for buffers with pH values between 4.5 and 9.2, Fig. 6(a). The plot of *OCP* values as a function of pH was linear on the whole range (R^2^ = 0.995), Fig. 6(d), following Eq. (4):

4 $$OCP/{\rm{m}}{\rm{V}}\,(vs.{\rm{S}}{\rm{C}}{\rm{E}})=+666.5\mbox{--}59.3\,{\rm{p}}{\rm{H}}$$

The slope of the line, −59.3 mV per pH unit (s.d. = 1.25), was close to the theoretical value predicted by Nernst’s equation for the electrochemical half reaction in Eq. (1). The PdO/Pd/Au/PMMA/PET electrode showed very good reversibility especially in acid and neutral media. For alkaline solutions a hysteresis of about 10 to 18 mV was registered.

The response of the PdO/Pd/Au/PMMA/PET electrode was also investigated in artificial sweat by measuring the *OCP* values in a titration continuous mode during approximately 120 s, Fig. 6(b). Small aliquots of NH_4_(OH) were added to the starting solution and the pH of the solution was confirmed by simultaneous measurement with a commercial pH sensor. The transition between consecutive pH values was evaluated by calculating the response time *t*_90_ < 30 s as the time required to achieve 90% change (Δ*OCP*_90_) from an initial *OCP* value to the final *OCP* value upon consecutive titrations, Fig. 6(c).

Also, the plot of *OCP* values function of pH was linear (R^2^ = 0.945) especially for pH values corresponding to mild acid and neutral media, Fig. 6(d), following Eq. (5)

5 $${OCP}/{\rm{mV}}(vs\cdot {\rm{SCE}})=+\,734.9\mbox{--}59.7\,{\rm{pH}}$$

The slope of the line, −59.7 mV per pH unit (s.d. = 16.41), confirmed the theoretical prediction. Nevertheless, deviation from linearity was observed for pH > 8.0.

### Proof of concept

In order to prove the concept of a functional wearable and flexible pH-sensor at epidermal level, an Ag/AgCl reference electrode was also produced on Au/PMMA/PET substrate, *Section 4 – Proof of concept*. Several architectures for the assembly of the working PdO/Pd/Au/PMMA/PET and the reference AgCl/Ag/Au/PMMA/PET electrodes were tested and the best response was obtained using the procedure described in Fig. 1(a). The PdO/Pd/Au/PMMA/PET pH sensor was placed on top of the AgCl/Ag/Au/PMMA/PET reference, with both sensing surfaces in top position but in an anti-parallel configuration while the gold covered extremity of each electrode provided the electric contacts with the potentiostat, Fig. 1(a). The aperture in the PdO/Pd/Au/PMMA/PET pH sensor allowed the electrochemical communication. At last, the sensor surface was coated with a polyacrylamide hydrogel that played a triple role: captured sweat, transported the analyte to the sensitive component and protected the sensor surface from the environmental factors ensuring a controlled medium. The hydrogel film was thin enough providing a short path to the electronic part components, ensuring a fast response time of the sensor.

The pH-sensor response was investigated by incubation in artificial sweat with different pH values as described in *Section 4 – pH sensor response in artificial sweat*. The *OCP* values function of time and of pH are plotted in Fig. 6(e). The results showed a fast and stable response time after incubation with a maximal drift of 3 mV min^−1^. The plot of *OCP* function of pH was linear (R^2^ = 0.921), Fig. 6(f), following Eq. (6)

6 $$OCP/{\rm{mV}}=+338.8\mbox{--}18.0\,{\rm{pH}}$$

The slope of the line, −18.0 mV per pH unit with a standard deviation s.d. = 9.37, was below the theoretical prediction. This is partially due to the complexity of the matrix of artificial sweat as demonstrated in *Section* 3.*2* and Fig. 6(d), but also to the low ionic mobility within the solid hydrogel electrolyte, which has been previously reported^9^.

The temperature dependence was also investigated. The OCP values were measured in artificial sweat pH = 7.16 at 25, 37 and 45 °C for at least 5 min. The dependence was linear and the average slope of the line was aprox. 1 mV/°C.

OCP values were also measured in artificial sweat after consecutive additions of 500 nL of HCl or NaOH. The minimum volume to which the sensor responses was found as the volume that gave rise to an increase in OCP three times the baseline noise level, and was calculated from LOD = 3 × S.D. × (sensitivity)^−1^. Using the equation *OCP*/mV = 216.47 + 0.11 × *V*/nL for titrations with HCl, the obtained value was approx. 250 nanoliters.

The mechanical resistance of the dried sensor was investigated by bending the device until the ends touch each other as shown in Fig. S1(a). OCP values were measured at pH = 7.16 after 10 and 100 consecutive bending cycles with s.d. ∼1 mV (Fig. S1(b)). The device was also investigated by scanning electron microscopy in terms of bending induced changes in micromorphology. No cracks into the dried hydrogel film were observed, the images being similar to those obtained for the initial state.

The sensitivity of the pH-sensor based on PdO/Pd/Au/PMMA/PET electrodes was compared with that obtained at other wearable sensors, essentially with electrochemical and electrical/electronic detection (see Table 3). It can be observed that with few exceptions, where sensitivities higher than 60 mV/pH unit were obtain with complicated electronic components and reading processes, the sensor developed in this study uses scalable, economic and reliable technologies, thus meeting all requirements needed for further development into a final, eventually commercial product.

**Table 3:** Comparison of the performance of some pH-sensors sensors reported in literature after 2016.

electrodes	transduction	sensitivity (mV/pH unit)	reference
PANi/PEDOT:PSS/MWCNT/cotton yarn	electrochemical	−61.0	^30^
HfO_2_/SiO_2_/Si	electronic	+36.0	^31^
SiOx/Al2O3//InGaZnO	electronic	+240.0*	^32^
graphite-polyuretane/graphene/PDMS	electrochemical	+11.3	^33^
IrO_2_/fabrics	electrochemical	−47.2	^34^
PANi/Au	electrochemical	−62.5	^35^
Au mesh/Au-doped graphene	electrochemical	−80.0	^36^
PEDOT:dye/oFET	electronic	∼100.0	^37^
PdO/Pd/Au/PMMA/PET	electrochemical	−18.0	this work

*At 100 accumulation cycles; PANi - polyaniline; PEDOT:PSS - poly(3,4-ethylenedioxythiophene) polystyrene sulfonate; oFET - organic field effect transistor.

## Conclusions

Quantification of H^+^ ions in biological fluids is useful for monitoring biological processes or disease diagnosis and treatment. The present work described the development of a flexible pH-sensor that can be integrated in wearable and non-invasive devices at epidermal level.

Poly(methyl methacrylate) electrospun fibers, coated with a layer of palladium/palladium oxide as sensing interface or with silver/silver chloride as reference electrode were employed successfully in developing such a highly flexible sensor.

Morphological and structural modifications of the sensing layer interface upon basic and acid treatment were characterized by scanning and transmission electron microscopy, and by X-ray photoelectron spectroscopy. The response to H^+^ ions was investigated by cyclic voltammetry and electrochemical impedance spectroscopy. The sensitivity of the electrodes was approx. −59 mV per pH unit determined by measuring the open circuit potential in different media with different pH values.

The functionality of the pH-sensor at epidermal level was proved by integrating the palladium/palladium oxide working electrode and silver/silver chloride reference electrode within a matrix of polyacrylamide hydrogel. Measurements were performed in artificial sweat The performance of the sensor was investigated in terms of sensitivity (up to −28 mV per pH unit), response time, temperature dependence (aprox. 1 mV/°C) as well as the volume of detection (250 nL) and mechanical resistance to bending. The present work addresses the sensing element of the wearable sensor. Further work will be performed in order to solve further challenges such as reliable contacts and readout component in such a way that the main characteristics of the device remain its low cost and flexibility/wearability.

## Supplementary information

Suplementary

## Data Availability

The datasets generated and analyzed during the current study are available from the corresponding author on reasonable request.
