# Supplementary material for: Palladium/palladium oxide coated electrospun fibers for wearable sweat pH-sensors
Source: Sci Rep. 2019 Jun 20;9:8902. doi: 10.1038/s41598-019-45399-2 (PMC6586619; doi:10.1038/s41598-019-45399-2)
Supplement: Supplementary file 1 — Suplementary [file 41598_2019_45399_MOESM1_ESM.pdf]

Supplementary information

Palladium/palladium oxide coated electrospun fibers for wearable sweat pH-sensors

Victor C. Diculescu, Mihaela Beregoi, Alexandru Evanghelidis, Raluca F. Negrea, Nicoleta G. Apostol, Ionut Enculescu

Photograph obtained during the mechanical stress test through bending of the pH-sensor and OCP values recorded before and after some bending cycles.

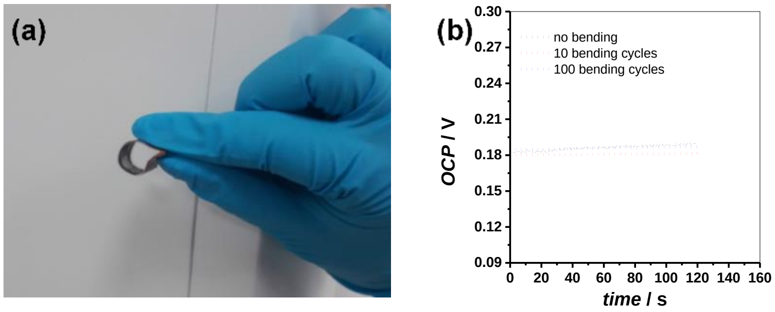

Figure S1. **(a)** Photograph of the sensor subjected to mechanical stress.

(b) OCP values of the pH-sensor recorded in artificial sweat at pH = 7.4 before and after 10 or 100 bending cycles.
